# Cardiac surgery in patients with previous pneumonectomy

**DOI:** 10.1186/1749-8090-3-11

**Published:** 2008-03-01

**Authors:** Sanjay V Ghotkar, Vikram Aerra, Neeraj Mediratta

**Affiliations:** 1 Department of Cariothoracic Surgery, Cardiothoracic Centre, Thomas Drive, Liverpool, UK

## Abstract

Severe pulmonary dysfunction is a commonly occurring postoperative complication following cardiac surgery. Resection of a lung causes major anatomical and physiological changes. Shift of the mediastinum and reduction in respiratory function following pneumonectomy makes cardiac surgery challenging not only for the surgeon but also for the anaesthetist. With improvement in life expectancy and better results following cardiac and pulmonary operations increasing number of patients are likely to be subjected to both of these operations during their lifetime.

There is paucity of data in the literature on the subject of cardiac surgery subsequent to previous pneumonectomy. We report our experience on performing cardiac surgery following pneumonectomy to highlight certain important features that we think are important while managing these patients.

## Background

Excision of lung entails significant anatomical changes in the rigid thoracic cavity. Typically the pneumonectomy space contracts by shift of mediastinum, elevation of diaphragm and crowding of ribs on the side of pneumonectomy. These secondary changes can make surgical access to mediastinal structures through the median sternotomy awkward and difficult. Pneumonectomy also reduces respiratory function by 45–55% and although the contra-lateral lung undergoes compensatory hypertrophy the respiratory reserve remains significantly diminished.

It is well known that major thoracic and abdominal surgery reduces the respiratory reserve significantly [1,2]. On the first postoperative day following sternotomy for a cardiac procedure, the observed decrement in forced vital capacity (FVC) is reported to be around 70% of the preoperative value. Ten days after surgery, when most patients can be discharged from the hospital, the FVC has increased but still remains 30% lower than the preoperative value [3-5]. There are reports stating deterioration of pulmonary function up to 3.5 months after cardiac surgery [3]. Because of this decline in the pulmonary function in the post-operative period the patients who have undergone a pneumonectomy would tolerate the cardiac surgery with difficulty.

There is scarcity of data in the literature on the subject of cardiac surgery subsequent to previous pneumonectomy [6,7]. We discuss our experience and review the literature on performing cardiac surgery following pneumonectomy to highlight technical and other features that need to be considered during management of these patients.

Our first patient was a 71-year male who had undergone intra-pericardial pneumonectomy 18 years earlier for squamous cell carcinoma of the left lung. His coronary angiogram revealed severe stenosis in left main stem and further significant disease in right coronary artery. He underwent urgent coronary artery bypass grafting (CABG) using cardiopulmonary bypass and antegrade cold blood cardioplegia. Saphenous vein (SVG) was used to graft left anterior descending artery (LAD) and posterior descending branch of the right coronary artery.

The left pneumonectomy had left this patient with paralysis of left vocal cord. Preoperative spirometry showed FVC 1.8 L (53% of predicted), FEV1 1.1 L (42% of predicted), FEV1/FVC ratio 79%. The postoperative course was complicated by excessive bleeding requiring re-exploration of chest, the cause of the bleeding was believed to be thromboasthenia as antiplatelet therapy including Aspirin and Clopidogrel was continued until the day of surgery due to severe nature of stenosis in left main coronary artery. On the second postoperative day this patient went in to atrial fibrillation and showed evidence of basal atelactasis on chest radiogram. Coughing efforts were poor and to avoid sputum retention in the solitary residual lung a mini-tracheostomy was performed to help expectoration. The subsequent recovery was slow but progressive and he was discharged home on 17^th ^postoperative day.

The second patient was a 77-year-old female who was diagnosed with severe aortic stenosis, she had undergone pneumonectomy 13 month earlier for squamous cell carcinoma involving right upper lobe. She underwent aortic valve replacement using a porcine bioproshthesis. Preoperative spirometry showed FVC 0.9 L (63% of predicted), FEV1 0.7 L (64% of predicted), FEV1/FVC ratio 104%. The postoperative recovery in her case was without any complications.

### Preoperative work up

In addition to routine blood test and spirometry the pre-operative workup of these patients included computerised tomography of the chest to assess distortion of intra-thoracic anatomy. Preoperative inspiratory muscle training is found to be beneficial reducing the incidence of postoperative pulmonary complications following thoracic and upper abdominal surgery [1,2,8,9]. In an attempt to improve the pulmonary function both the patients were admitted prior to their surgery for intensive chest physiotherapy and incentive spirometry.

## Discussion

Following the pneumonectomy access to the heart is more difficult as heart not only shifts towards the side of pneumonectomy but also posteriorly due to loss of it's anchor to pulmonary veins. Following right pneumonectomy, left to right displacement of the mediastinum occurs mostly by transfer with subsequent dextroposition of the heart and arrangement of the aortic arch in a frontal plane. On the contrary, after left pneumonectomy right to left shift occurs mostly through rotation with the aortic arch arranged in the sagital plane [10]. Severe distortion of mediastinal anatomy following pneumonectomy has been reported in the literature. [11-13]

While performing the operation care has to be taken during sternotomy to avoid injury to the hyperinflated lung that often lies in close contact with posterior surface of the sternum. We encountered intrapericardial adhesions on the posterior surface of the heart in our first patient this, we believe, occurred due to intrapericardial technique used while performing pneumonectomy on this patient.

Following right pneumonectomy the shift of the heart in the right hemithorax can make access to right atrium for establishing cardiopulmonary bypass (CPB) difficult. In their case report, Berrizbeitia et al report difficulty they encountered during cannulation of inferior venacava (IVC) in a patient who had undergone right pneumonectomy [6]. The fixity of IVC at it's hiatus in diaphragm prevents it from moving rightward resulting in acute angulation between right atrium and the IVC. The venous cannulation to establish CPB may be achieved with more ease if operator stands on left side of the patient. Another factor that needs to be considered in a patient who had previous right pneumonectomy is that, right superior pulmonary vein is not available for venting therefore, alternative sites may have to be used.

Following left pneumonectomy access to arteries in the circumflex region becomes difficult due to shift of heart to the left side, this difficulty has also been reported by Medalion et al [7]. We did not come across any report of off pump coronary artery bypass graft surgery (OPCAB) being performed on patients with previous pneumonectomy, but we estimate that following left pneumonectomy, exposure of posterior surface of heart to graft circumflex coronary artery territory during an OPCAB procedure would be technically challenging.

We would like to highlight certain important points that need to be considered while choosing internal thoracic artery (ITA) as a conduit while performing CABG in these patients. The chest wall on the side of pneumonectomy becomes rigid, hence spreading of sternal retractor leads to uneven spreading. ITA from the side of pneumonectomy is difficult to harvest. Pedicled ITA may not reach its targeted vessel due to displacement of heart moreover; the ITA graft may become kinked by the hyper-inflated lung. Various studies have shown that increased pain associated with ITA harvesting may be responsible for decreased postoperative pulmonary function [14-16]. This becomes more relevant in patients who already have compromised pulmonary function. Demirtas et al have reported use of left ITA to graft LAD in a patient who had left pneumonectomy in the past [16]. Their patient developed acute left heart failure on arrival to postoperative intensive care unit and required internal cardiac massage. Berrizbeitia et al used SVG to graft LAD[6]. We did not use the ITA in our patient who had undergone left pneumonectomy.

There are important considerations for the anaesthesiologist too, internal jugular vein cannulation can be difficult following pneumonectomy due to shift of the mediastinum. Care has to be taken to protect the single lung and sudden fluid overload can lead to pulmonary oedema [11]. Early extubation is desirable to avoid risk of prolonged ventilation; external warming at the cessation of CPB and on arrival to intensive care unit could facilitate early extubation. Use of thoracic epidural analgesia improves pain control and facilitates easy expectoration and avoids many of the post-operative pulmonary complications [17,18]. Similarly, intensive chest physiotherapy and early mobilization becomes more important in these patients to avoid atelactasis and risk of deep vein thrombosis. We utilised benefits of epidural analgesia and stressed on early mobilisation and chest physiotherapy in both our patients.

## Conclusion

We conclude that with attention to the specific features of the preoperative, intraoperative, and postoperative management, open heart procedures can be performed successfully on patients after pneumonectomy.
